# Design of high-sensitivity La-doped ZnO sensors for CO_2_ gas detection at room temperature

**DOI:** 10.1038/s41598-023-45196-y

**Published:** 2023-10-26

**Authors:** Khaled Abdelkarem, Rana Saad, Adel M. El Sayed, M. I. Fathy, Mohamed Shaban, Hany Hamdy

**Affiliations:** 1 Nanophotonics and Applications (NPA) Lab, Department of Physics, Faculty of Science, Beni-Suef University, Beni-Suef, 62514 Egypt; 2 Physics Department, Faculty of Science, Fayoum University, El Fayoum, 63514 Egypt; 3 Department of Physics, Faculty of Science, Islamic University of Madinah, 42351 Madinah, Saudi Arabia

**Keywords:** Materials science, Materials for devices, Nanoscience and technology, Nanoscale devices, Nanoscale materials, Techniques and instrumentation

## Abstract

For the sake of people's health and the safety of the environment, more efforts should be directed towards the fabrication of gas sensors that can operate effectively at room temperature (RT). In this context, increased attention has been paid to developing gas sensors based on rare-earth (RE)-doped transparent conducting oxides (TCO). In this report, lanthanum-doped zinc oxide (La-doped ZnO) films were fabricated by sol–gel and spin-coating techniques. XRD analysis revealed the hexagonal structure of the ZnO films, with preferred growth along the (002) direction. The crystallite size was decreased from 33.21 to 26.41 nm with increasing La content to 4.0 at.%. The UV–vis–NIR indicating that the films are highly transparent (˃ 80%), La-doping increased the UV blocking ability of the films and narrowed the optical band gap (Eg) from 3.275 to 3.125 eV. Additionally, La-doping has influenced the refractive index of the samples. Gas sensing measurements were performed at ambient temperature (30 °C) and a relative humidity (RH) of 30%, employing different flow rates of carbon dioxide (CO_2_) gas used synthetically with air. Among the evaluated sensors, the ZnO: 4.0 at.% La sensor exhibited the most significant gas response, with a value of 114.22%. This response was observed when the sensor was subjected to a flow rate of 200 SCCM of CO_2_ gas. Additionally, the sensor revealed a response time of 24.4 s and a recovery time of 44 s. The exceptional performance exhibited by the sensor makes it very appropriate for a wide range of industrial applications. Additionally, we assessed the effect of humidity, selectivity, reusability, repeatability, detection limit, and limit of quantification.

## Introduction

Gas sensors play a crucial role in ensuring a safe environment. Gas level monitoring and control systems play a crucial role in ensuring human safety and environmental protection. Gas sensors are crucial in detecting hazardous gases like carbon monoxide (CO), carbon dioxide (CO_2_), and nitrogen dioxide (NO_2_) to prevent life-threatening situations^1,2^. Early detection is crucial for protecting human lives. Also, gas sensors play a vital role in environmental conservation. Gas sensors can monitor emissions from various sources, such as industrial processes and transportation, to ensure compliance with environmental regulations and minimize the impact on the ecosystem. CO_2_ gas sensors are necessary for monitoring and maintaining healthy indoor air quality, as high levels of CO_2_ can have detrimental effects on human health, and for prompting adjustments in ventilation and heating, ventilation, and air conditioning (HVAC) systems. In addition, CO_2_ sensors play a role in mitigating climate change by monitoring emissions and identifying sources of excessive CO_2_ release in industrial processes. This data assists organizations in implementing mitigation strategies, decreasing their carbon footprint, and contributing to global initiatives aimed at addressing climate change^3^. Due to the influence of carbon dioxide (CO_2_) emissions on global warming and daily life, the CO_2_ sensors operating at RT became essential for a safe environment and monitoring the quality of indoor air^4–8^. The fabrication of gas sensors based on transparent conducting oxides such as ZnO has attracted the interest of several researchers^9,10^. Besides the eco-friendliness, ease of fabrication, and abundance in nature, ZnO exhibits distinctive advantages represented in the hexagonal structure, band gap in the range of 3.26–3.37 eV, and exciton binding energy of about 60 meV^11–13^. H.-Rivera et al.^14^ reported the possible use of Cu-doped ZnO, prepared by the co-precipitation method, as a gas sensor for propane of 500 ppm concentration but at a high temperature (300 °C). Doping ZnO with rare-earth (RE) elements for gas sensing applications has attracted increased attention. RE oxides have a large surface area, improved ability towards charge carrier trapping, and thus can greatly reduce exciton recombinations. Among RE ions, La‏^3+^ can narrow the photonic gap and produce oxygen vacancies to increase the separation between electrons and holes^15^.

Lu and Zhu^16^ prepared Zn_0.97_La_0.015_Ce_0.015_O rods, 2–5 μm in length and 100–200 nm in diameter, by the mild-hydrothermal method, which possessed a response and recovery features of 11 and 45 s towards 100 ppm of 1,2-propanediol at 240 °C, respectively. Li et al.^17^ prepared La-doped ZnO plates, 500 nm in diameter and 50 nm in thickness. They reported a gas sensing response for the sample doped with 3.0 at.% La to acetone (200 ppm at 330 °C), and ethanol (120–200 ppm at 300 °C). Shingange et al.^18^ developed La-doped ZnO nanofibers by using the electrospinning method. 2.0 at.% La doping and annealing at 900 °C resulted in the highest H_2_S response with good repeatability and stability. At this temperature, the formed *p*-type La_2_O_3_ mixed with the *n*-type ZnO played a significant role in enhancing the H_2_S sensing feature. Santhosam et al.^19^ prepared Zn_1-x_La_x_O (x = 0, 1, 3, 5 at.%) by spray technique and reported fast response and recovery times of 39 and 11 s at 100 ppm of NH_3_ at RT.

ZnO is employed in gas sensors for the purpose of CO_2_ detection. The distinctive characteristics of this device make it well-suited for the detection of CO_2_ gas. ZnO has a pronounced reactivity towards gases such as CO_2,_ leading to significant alterations in its electrical properties. The heightened sensitivity of this technology enables accurate detection and measurement of CO_2_ levels in ambient air or confined spaces. ZnO possesses the advantageous characteristics of being both inexpensive and readily available, rendering it a financially viable alternative for the production of CO_2_ sensors on a wide scale. ZnO possesses the characteristics of being non-toxic and environmentally benign, rendering it highly suitable for applications in indoor air quality monitoring and the mitigation of greenhouse gas emissions. The long-term stability and reliability of ZnO-based CO_2_ sensors are crucial for continuous monitoring applications. ZnO enables the monitoring of CO_2_ levels in real-time, exhibiting rapid response and recovery capabilities. The versatility of this technology allows for its broader utilization in other domains, such as wearable sensors, smart buildings, and Internet of Things (IoT) devices, hence facilitating enhanced monitoring of CO_2_ levels. ZnO can also be adjusted to enhance its selectivity towards CO_2_, thereby minimizing the impact of external factors. In summary, the sensitivity, cost-effectiveness, environmental compatibility, stability, and integration capabilities of ZnO render it an indispensable material for the advancement of efficient CO_2_ gas sensing systems aimed at monitoring and mitigating CO_2_ emissions^13,20,21^. This article investigates how lanthanum-doped zinc oxide (La-doped ZnO) gas sensors could be used in many different fields. This novel gas sensing method detects CO_2_, addressing air quality, climate change, and industrial emission issues. This system operates at room temperature, eliminating the need for energy-consuming heating devices and enhancing sensor reliability and energy economy. Gas sensors are used in industries, chemical processing, and healthcare. They are essential for real-time gas concentration monitoring for process optimization and safety. Transparent conducting oxides (TCOs) in this cost-effective technology may reduce installation and maintenance costs. This makes it appealing for study and practice. This work also advances the science of rare-earth-doped TCOs' optical and gas-sensing features. Additionally, it advances materials science and gas sensing technologies and offers a diverse and impactful solution for numerous businesses and scientific endeavors. The present investigation involved the synthesis of hexagonal thin films of ZnO and La-doped ZnO by utilization the spin coating technique. The investigation focused on examining the morphological structure, chemical composition, and optical properties of thin films made of pure ZnO and ZnO doped with La. Furthermore, an investigation was conducted on the gas sensing mechanism of CO_2_, wherein many parameters, including surface roughness, band gap size, and particle size, were shown to exert influence.

## Materials and experimental method

### Materials and films preparation

Pure and Zn_1_-_x_La_x_O (x = 0.0, 0.02, and 0.04) thin films were prepared by sol–gel, followed by spin-coating and annealing. A 0.5 M solution of Zn(CH_3_COO)_2_·2H_2_O, (molecular weight M_W_ ~ 219.5 g/mol, supplied by Panreac) was prepared by dissolving the required mass in 10 ml of 2-methoxy ethanol of M_W_ ~ 76 g/mol. Then the monoethanol amine (MW ~ 61 g/mol, supplied by Scharlab S.L., Spain) was used to stabilize the Zn ions and added at a 0.5M concentration. The resulting solution was stirred at ~ 50–55 °C for 2 h to be clear and homogenous, and aged at RT for more than 20 h before the spin-coating process. The source of La^3+^ ions was LaCl_3_ heptahydrate (M_W_ ~ 371.4, supplied by Schorlau, Spin) that was added at ratios of 2.0 and 4.0 at.%. The deposition was carried out on glass substrates pre-cleaned in an ultrasonic bath using acetone, then ethanol, and finally deionized water. Moreover, the glass substrates were dried with an air gun before use. Each film was spin-coated at an rpm of 2000 for a period of 30 s. Each layer was dried at ~ 180 °C for 15 min. The coating and drying process was repeated six times to obtain films with a reasonable thickness. The pure and La-doped films were annealed at ~ 500–525 °C for 2 h in an air furnace.

### Characterization techniques and evaluating CO_2_ gas sensing ability

**Figure 1:**
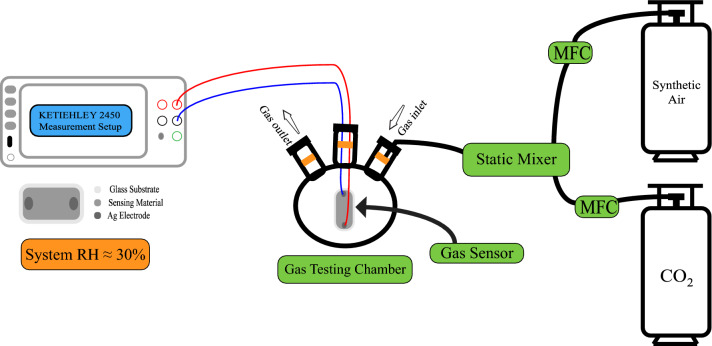
A schematic depiction of the gas detecting and measurement system.

To evaluate the crystalline phase, crystallite size, and other structural parameters, a high-resolution X-ray diffractometer (Philips X'Pert Pro MRD) with Cu K_α_ radiation of wavelength *λ* = 1.5418Å and a step of 0.02**°** was used. The UV–vis-NIR optical spectra in the mode of reflectance and transmittance, in the range of 200–1100 nm, were recorded at RT on a UV/VIS/NIR 3700/double beam spectrophotometer supplied by Shimadzu. The integrated sphere optical component and barium-petroleum sulfonate were used as references to provide a nominal 100% reflectance measurement. The films' morphology and chemical composition were investigated using field emission-scanning electron microscopy (FE-SEM: ZEISS/SUPRA/55/VP and ZEISS/LEO/Gemini Column) and an energy dispersive X-ray unit (EDX) supplied by Oxford Link ISIS/300/EDX. As illustrated in Fig. 1, the gas sensing measurement system employed a conventional measurement circuit designed for commercially available metal-oxide gas sensors. A 1.0-L three-neck round-bottom flask, which was outfitted with rubber O-rings at the upper ends of its three necks, was produced for the purpose of conducting gas sensing tests. The flask included three distinct components: a gas input neck, an exit neck, and an electrical signal receiving neck. A 100% CO_2_ gas sample was obtained from a gas cylinder supplied by the Beni-Suef Factory for medical and industrial gases, with synthetic air used in the flask. The gas flow rate was monitored via an Alicat MC-500SCCM-D gas mass flow controller, and the CO_2_ gas was subsequently blended with air by a synthetic process. Thin films at both ends of the sample were coated with a thin layer of Ag and connected to the Keithley measurement-source device (model 2450) via copper wires to collect charges^22^. The experimental setup was kept at a constant temperature of 30 °C during the whole data collection procedure, and the relative RH was 30%. Ohmic connections were formed by employing conductive silver paste as the electrodes. The voltages of the sensor were monitored and examined subsequent to the adjustment of the quantity of carbon dioxide delivered into the flask. In order to conduct the selectivity test, gas cylinders of high purity were procured, each containing CO_2_, CO, NO_2_, and H_2_S.

## Result and discussion

### Characterization

#### X-ray diffraction analysis

**Figure 2:**
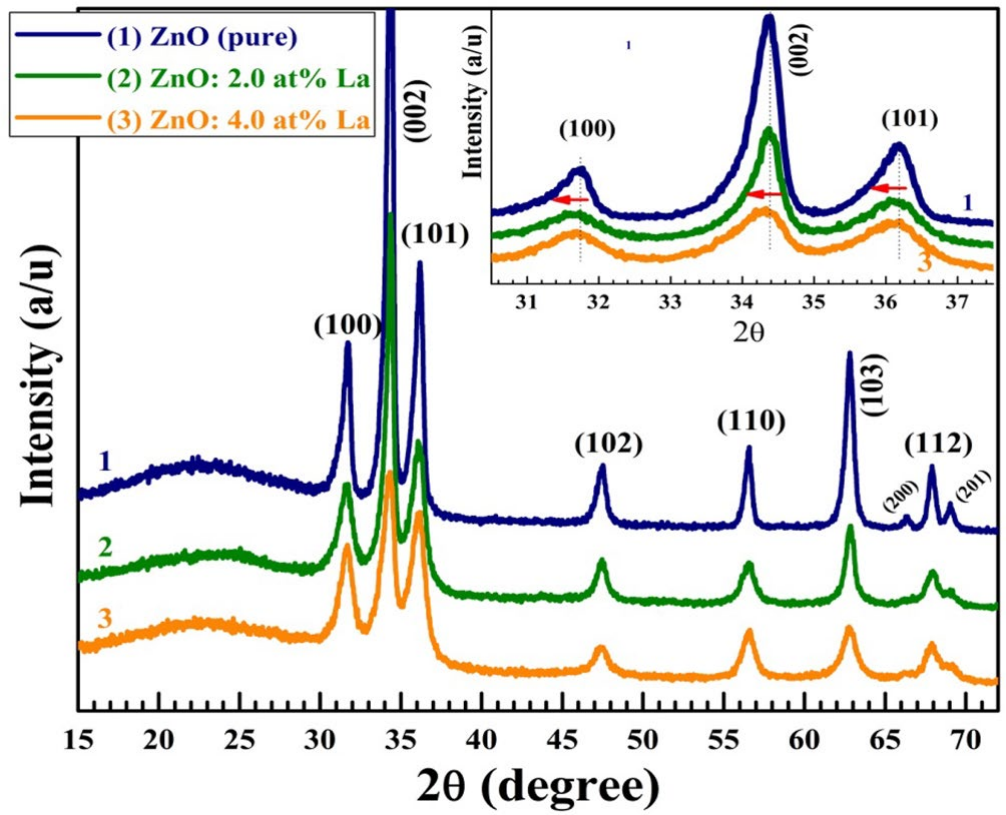
X-ray diffraction of pure and La-doped ZnO spin-coated films.

X-ray diffraction (XRD) was applied to investigate the crystalline phase of ZnO and La-doped ZnO thin films and to calculate the average crystallite size (*D*). As shown in Fig. 2, the patterns illustrate the successful formation of the hexagonal (wurtzite) crystal phase of ZnO according to JCPDS 01–089-0510. For comparison, the XRD data of pure and ZnO: 4.0 at% La are shown in Fig. S1, see the supplementary materials file. No peaks related to La or La_2_O_3_ can be detected in the XRD patterns, which indicates the successful incorporation of La inside ZnO lattices. 3.0 at.% Ce or La doping resulted in CeO_2_ and La_2_O_3_ phase formation in ZnO rods prepared by the mild-hydrothermal method^16^. In addition, Li et al.^17^ detected the La_2_O_3_ phase in the 2.0 at.% La-doped ZnO plates prepared by the hydrothermal method. This means that sol–gel combined with the spin-coating technique is more relevant for rare earth-doped ZnO thin films. The values of *D* were determined using the Scherer equation: $D= \frac{0.9 \mathrm{x }0.154 nm}{ FWHM \mathrm{xcos}\theta }$, where the numerator (0.9 × 1.54) is the Scherer constant multiplied by the wavelength of the used Cu ${K}_{\alpha }$ radiation, and FWHM is the full-width-at-half-maximum intensity^9^. Considering the most intense three peaks (100), (002), and (101), the *D*_av_ of the pure ZnO is 33.21 nm, decreasing with increasing La ratio until 26.41 nm at 4.0 at.% La, as listed in Table 1. The values of the following parameters are also listed in Table 1: the peak position (2θ), FWIM, relative intensity (*I*/*I*_o_), and the values of the texture coefficient ${T}_{\mathrm{C}}=(I/{I}_{\mathrm{o}})/[{N}^{-1}\sum \frac{I}{{I}_{\mathrm{o}}}]$, where *I*_o_ is the standard intensity of each plane as given from the JCPDS data and *N* is the number of reflections^23,24^. The values of *T*_C_ indicate the presence of preferred growth; the crystals are more oriented along the (002) direction in all films. This predominance of (002) along the *c*-axis is not influenced by La incorporation at 2.0–4.0%. This indicates the possibility of utilizing the films in a piezoelectric application. As seen in the inset of Fig. 2, the main three peaks were shifted to lower 2θ values after doping with 2.0–6.0 at.% La. The substitution of Zn (ionic radius = 0.74 Ǻ) with the greater ionic radius of La (1.16 Ǻ) significantly distorted the ZnO crystal lattice. According to Bragg's law of *d*-spacing, α $\frac{1}{\mathrm{sin}\theta }$ implies that the films became more compact and denser after La incorporation^25^. A similar observation was reported for 1.0 at.% Cu or Ni into the ZnO lattice^14^ and for the hydrothermally prepared La-doped ZnO films^15^. However, the peaks of 0.0–2.0 at.% La-doped ZnO nanofibers shifted to left or to right depending on the annealing condition (500–900 °C) and the segregation of La_2_O_3_^18^.

**Table 1:** XRD parameters of ZnO (Pure), 2.0 at.% La, and 4.0 at.% La:

Sample	ZnO (pure)			2.0 at.% La			4.0 at.% La		
Parameter	(100)	(002)	(101)	(100)	(002)	(101)	(100)	(002)	(101)
2θ	31.752	34.436	36.250	31.735	34.433	36.245	31.629	34.395	36.235
FWHM	0.2952	0.3936	0.3542	0.451	0.5117	0.433	0.5133	0.3952	0.481
*I/I*_o_	24.58	100	39.93	60.09	100	85.09	24.89	100	34.57
*T*_C_	0.448	1.824	0.728	0.735	1.224	1.041	0.468	1.881	0.650
*D*_av_ (nm)	33.21			31.83			26.41		

#### Energy-dispersive X-ray spectrum

**Figure 3:**
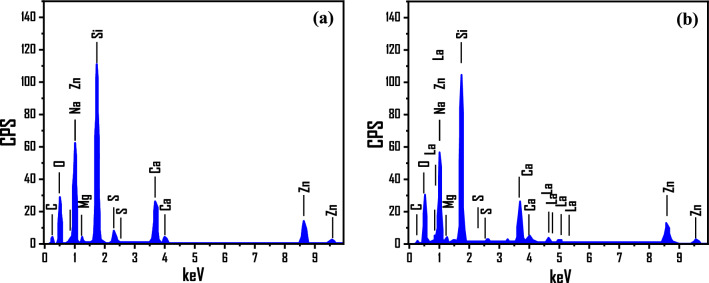
EDX of (**a**) pure ZnO, and (**b**) 4.0 at.% La-doped ZnO films.

Figure 3a,b depict qualitative and quantitative EDX evaluations of undoped and La-doped ZnO films, respectively. The undoped film exhibited a purity of 80.4% Zn and a stoichiometric Zn/O ratio of 19.6%. The Si, S, Ca, Mg, and Na signals are visible from the glass substrate because of the interaction volume (> 1 μm at 11 keV) > ZnO thickness. The Zn, O, and La signals for La-doped film were displayed in Fig. 3b, showing the quantitative incorporation of La interstitially in the ZnO lattice despite the difference in the atomic radii of La and Zn. These results are aligned with the XPS data shown in Fig. S2.

#### Scan electron microscopy (SEM) and surface roughness

**Figure 4:**
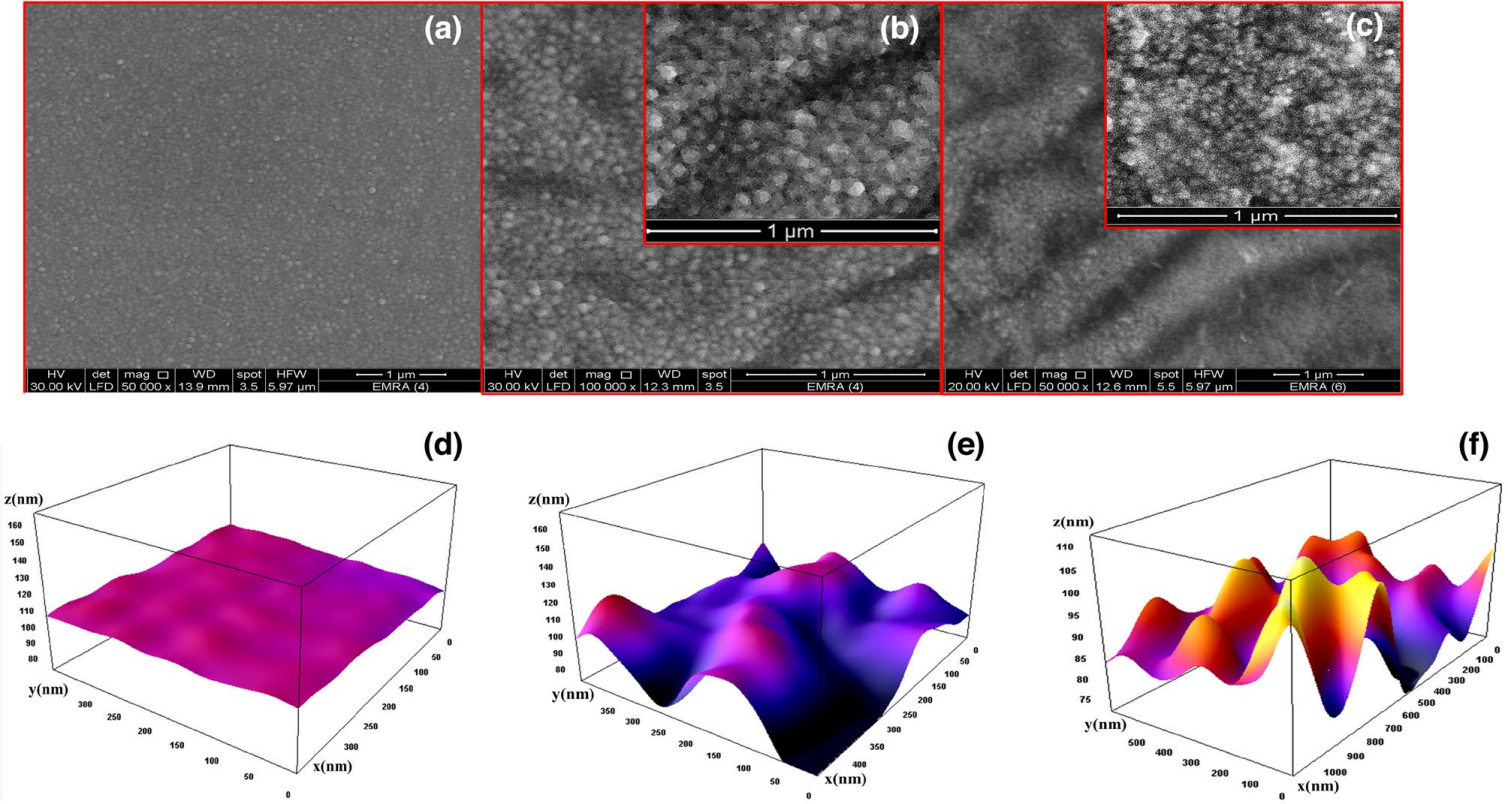
SEM images of (**a**) pure, (**b**) 2% La-doped, (**c**) 4% La-doped ZnO nanostructured thin films. Surface roughness for ZnO thin films,(**d**) pure, (**e**) 2% La-doped ZnO and (**f**) 4% La-doped ZnO.

Figure 4 shows FE-SEM images of the surface microstructure of undoped, 2% La-doped, and 4% La-doped ZnO films spin-deposited on glass substrates. When doped with La, the smooth surface of the un-doped film, Fig. 4a, transformed into a wrinkle net-work structure in the 2% La-doped film, Fig. 4b, and the resolution of this wrinkle net-work structure increased as the doping ratio climbed to 4% La, Fig. 4c. This might be explained by increased surface roughness with increasing La concentration^26^. Thus, the surface texture is determined by the ratio of the integrated element in the host material. Shelke et al. used spin coating to make pyramid-shaped Sn-doped ZnO films and In-doped ZnO with sand-dune-shaped nanostructures^27,28^. Used Na to modify the surface morphology of ZnO nanofilms to design efficient gas sensors. Shaban and El Sayed used codoping to control the surface morphologies of ZnO nanofilm^29,30^. The average film thickness for the pure and 2% La-doped Zno in Fig. S3 is approximately 351, and 315 nm, respectively. As a result, the average rate of growth of the film is 105 nm/min. The 2% La-doped film is formed by the aggregation of virtually spherical NPs with diameters smaller than 55 nm, as seen in the SEM picture in Fig. 4b. The density of the agglomerated particles rose with 4% La doping, while particle diameters fell to less than 40 nm. Similarly, Nd-doped ZnO films have shown comparable results^31^. As a result, the nanoparticles in these textured films have a narrow size distribution and a high density per unit area. As a consequence, the doped films can be used for sensing and photocatalysis. ImageJ software was used to measure the surface roughness of pure ZnO, 2% La-doped ZnO, and 4% La-doped ZnO thin films. The results pertaining to this are depicted in Fig. 4d–f. It is worth mentioning that the surface roughness of the thin film of ZnO doped with 4% La was found to be greater in comparison to both the ZnO films doped with 2% La and the undoped ZnO films. The increased development of a wrinkled network structure within the 4% La-doped ZnO coating is thought to be the cause of the increase in surface roughness that was seen. As a result, the enhanced surface area that arises from the increased roughness plays a role in augmenting gas sensitivity because rough surfaces have a larger surface area compared to smooth surfaces. This increased surface area provides more active sites for gas molecules to adsorb, increasing the probability of gas-surface interactions. Consequently, the gas response, such as the adsorption or reaction of carbon dioxide, can be enhanced. The presence of surface roughness has the potential to alter the distribution of electric charge in the vicinity of the ZnO thin film surface. This change could make more surface states or defect sites available, which could then be used as active sites for gas adsorption or reactions. The existence of these active sites can augment the material's receptiveness to carbon dioxide, resulting in an amplified gas reaction^22,29^.

#### Optical properties

**Figure 5:**
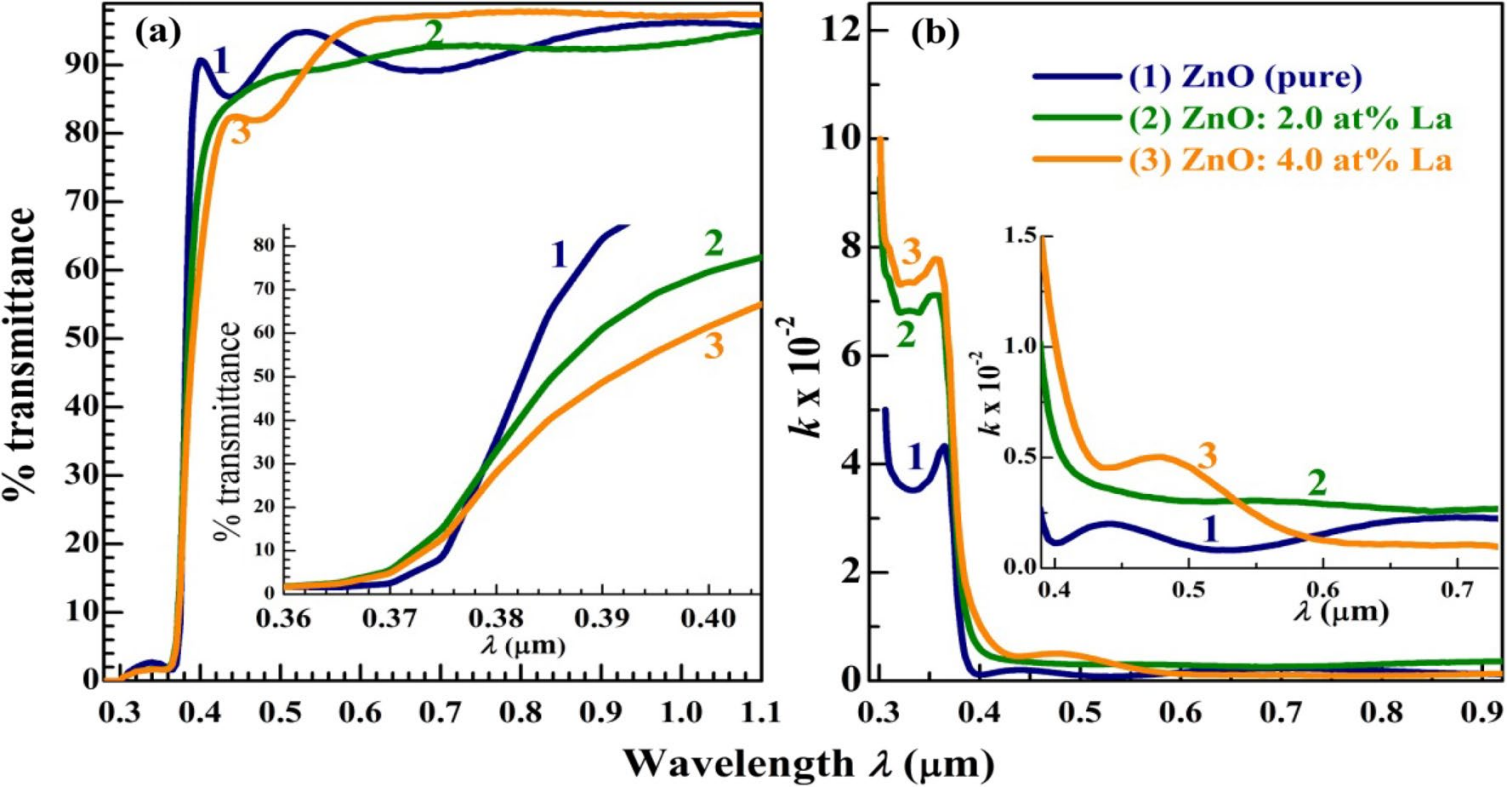
Optical properties of pure ZnO and 4% La-doped ZnO (**a**) absorbance and (**b**) transmittance, and (**c**) Plots of (αhν)^2^ versus hν to determine E_g_.

Figure 5a,b shows the transmittance spectra and extinction coefficient (*k*) of the pure and La-doped ZnO films after the glass substrate effect has been taken into account. The *k* values were determined from the equation: $k=\frac{\alpha \lambda }{4\pi }$, where α is the absorption coefficient, and from the transmittance spectra as $\alpha =\frac{1}{t}\mathrm{ln}\left(\frac{1}{T}\right)$, where t is the film thickness. The films exhibit a high transmittance (≥ 80%) in the visible and IR regions. The apparent interference fringes may result from the layered structure of the films originating due to the six spin-coated layers. The 4.0 at.% La-doped ZnO shows the lowest T% in the range of 378–540 nm and the highest T% at λ ˃ 580 nm. As seen in the inset of Fig. 5a, the absorption edge appears at λ ˂ 390 nm and shifts towards longer λ with increasing the La doping ratio. Figure 5b shows that the k values are very small (˂ 0.01) in the visible and IR regions and increase with La doping, especially in the UV region, from 0.04 to ~ 0.08. This indicates the enhancement of ZnO blocking ability towards UV radiation by La doping^32^.

Based on the optical absorption theory, Tauc's relation can take the following form^33^: $\alpha =\frac{{A(h\nu -{E}_{g})}^{b}}{h\nu }$, where A, hν, and Eg are a constants, the incident energy, and the optical band gap, respectively^34^. For the direct transition, b = 0.5, and hence ($\alpha h\nu {)}^{2}=A(h\nu -{E}_{g})$. Plotting (αhν)^2^ vs. hν gives the E_g_ by extending the linear portions of the curves to $\alpha$ = 0, as shown in Fig. 5a ^24,35,36^. The derived E _g_ are 3.275, 3.20, and 3.125 eV for ZnO pure, 2.0 at.%, and 4.0 at.% La-doped ZnO, respectively. This reduction in E_g_ value is due to La incorporation, the induced change of lattice structure, or distortion, observed from XRD data, associated with the generated defects, and the introduction of impurity levels in the band gap of ZnO. Similarly, Yb-, Er-, and La-doped ZnO nanorods, prepared by the co-precipitation method, reduced the Eg of ZnO from 3.26 eV to 3.22 and 3.21 eV^11^. In addition, in the sol–gel-prepared Ce- and La-doped ZnO nanoparticles, the Eg was reduced from 3.37 eV to 2.97 eV (at 1.2 at.% La) and 2.76 eV (at 2.0 at.% Ce)^12^.

The refractive index (n) of the films can be derived from the corrected reflectance (R) according to the relation^37^: $n=\frac{1+\sqrt{R}}{1-\sqrt{R}}.$ The variation of n with λ is depicted in Fig. 5b. The n exhibits oscillatory behaviour, and its values are in the range of 1.15–3.9. Similar phenomena were also detected in the spectra of Mg-doped SnO films^37^. According to the wave theory of light that accounts for the bending phenomena that cause the scattering, the decrease in particle size yields an increased amount of the first surface reflections, multiple scattering, and therefore the reflectivity and refractive index^32^.

### Gas sensing properties

#### I-V characteristic

**Figure 6:**
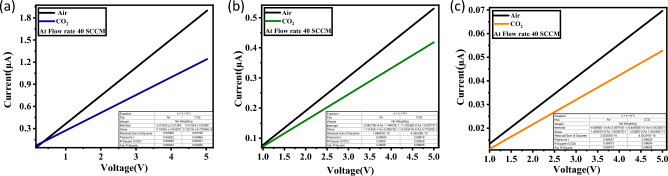
I–V curves of CuO thin films in the presence and absence of 40 SCCM of CO_2_ gas; (**a**) pure ZnO; (**b**) ZnO: 2.0 at% La; and (**c**) ZnO: 4.0 at% La at RT and 30% RH.

A comprehensive analysis was carried out to examine the influence of La doping concentration on the CO_2_-sensing properties of ZnO thin films at RT (30 °C). The voltage range of 0 to 5 V was used to examine the I-V characteristics of the ZnO films, as shown in Fig. 6. It is important to note that all of the thin films had a linear current–voltage (I-V) characteristic. This means that the electrodes and ZnO films formed reliable ohmic electrical connections. The aforementioned observation provides empirical evidence supporting the effective propagation of charge carriers within the sensing material, thereby guaranteeing continuous electrical conduction^38,39^.

The incorporation of La dopant into the ZnO films led to a decrease in the electrical conductivity of the devices. Additionally, as shown in Fig. 6, the presence of CO_2_ had an additional impact on the conductivity behavior. In particular, with a consistent voltage of 5 V, the current exhibited a reduction in each doped ZnO sample in comparison to the pure ZnO. In the case of pure ZnO (Fig. 6a, there was a reduction in current from 1.9 to 1.24 μA). Similarly, in ZnO: 2.0 at% La Fig. 6b, the current reduced from 0.53 to 0.41 μA. Furthermore, in ZnO: 4.0 at% La (c), the current decreased from 0.07 to 0.052 μA. The results indicate that the addition of La to ZnO resulted in an increase in sensitivity and gas reaction towards CO_2_, with the most significant impact found in ZnO containing 4.0 atomic percent of La. The observed behaviour can be attributed to the heightened surface roughness^39^, and the possibility that Lanthanum dopants may segregate or migrate to the surface of the ZnO thin film during exposure to CO_2_. Lanthanum has a larger ionic radius than zinc, and its separation can break up the crystal lattice, cause localized defects, or create energy barriers for the movement of charge carriers. These effects can increase the resistance of the film^40,41^.

#### Dynamic response

**Figure 7:**
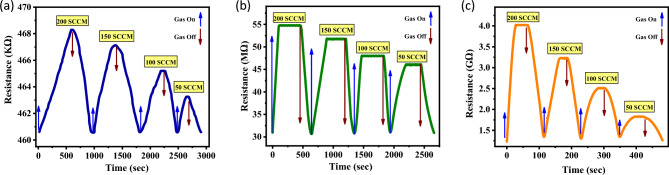
Illustrates the resistance response behavior of the ZnO sensors: (**a**) pure, (**b**) ZnO: 2.0 at.% La, and (**c**) ZnO: 4.0 at.% La at RT and 30% RH.

In the study, the synthesized sensitive layers were exposed to varying CO_2_ gas flow rates while being balanced with air at RT and at 30% RH. The data obtained from these tests was subjected to analysis. In Fig. 7, the dynamic resistance responses of undoped and lanthanum-doped ZnO films are depicted under balanced CO_2_ and air atmospheres at RT and 30% RH. The measurements were conducted at various flow rates of CO_2_, specifically 50, 100, 150, and 200 SCCM, while maintaining a balanced air mix. The alteration in the concentration of charge carriers on the ZnO sensors' surface, resulting from the interaction with CO_2_ gas, is responsible for the observed variation in sensor resistance. ZnO demonstrated n-type characteristics, signifying its ability to conduct electricity through the movement of negative electrons as the predominant charge carriers as opposed to positive holes. The observed rise in resistance of ZnO sensors when exposed to different flowrates of CO_2_ gas can be attributed to the oxidizing properties of CO_2_. This finding provides evidence for the n-type material response of both pure and La-doped ZnO. Hence, it was observed that the ZnO gas sensor exhibited a reduction in current and an increase in resistance upon exposure to non-polar and oxidizing CO_2_ gas molecules^13^. Figure 7 illustrates the impact of varying the CO_2_ flow rate, ranging from 50 to 200 SCCM at RT, on the gas responses of ZnO sensors. The resistance of the sensors exhibited a positive correlation with the CO_2_ flow rate, validating the effective functionality of the proposed sensors. After the gases were able to exit, the resistance of the sensors returned to its original value. According to the results shown in Fig. 7a, it was observed that the resistance of the pure ZnO thin film at 200 SCCM exhibited an increase from 460.6 to 468.3 KΩ. In a similar manner, the ZnO: 2.0 at% La sensor, as shown in Fig. 7b, exhibited a resistance increase from 30.9 to 54.7 MΩ. Also, the resistance of the ZnO: 4.0 at% La sensor exhibited an increase from 1.23 to 4 GΩ, as depicted in Fig. 7c. The observed rise in resistance can potentially be attributed to the interaction between CO_2_ and the surface of the ZnO thin film, resulting in the formation of passivating layers such as carbonates or oxides. The presence of passivating layers can serve as insulating barriers, impeding the mobility of charge carriers within the film and resulting in an elevation of resistance^42^.

#### Gas response, flow rate sensitivity, response time, and recovery time

**Figure 8:**
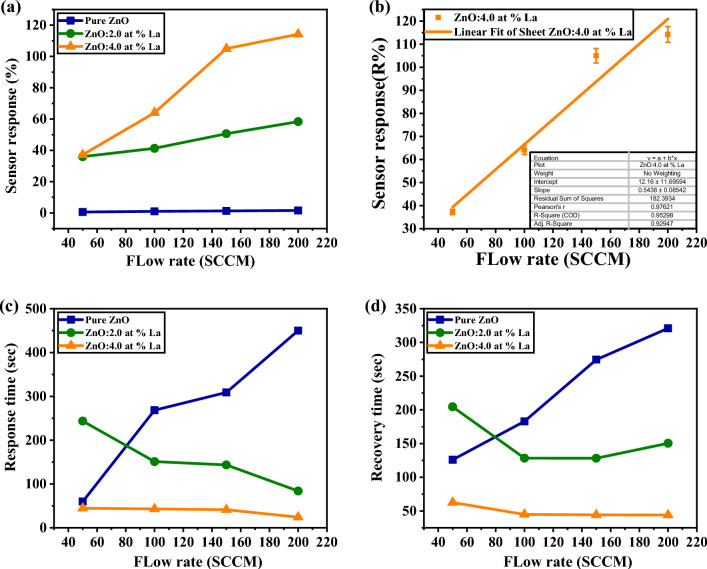
(**a**) The sensor gas response of pure and La-doped ZnO films, (**b**) linear fitted sensor response versus CO_2_ Flow rate, (**c**) the response time, and (**d**) the recovery time of ZnO thin films all vs different gas flow rates at RT and 30% RH.

The graphical representation in Fig. 8 establishes a correlation between the sensor response and the flow rate of CO_2_ gas, ranging from 50 to 200 SCCM. The sensor response (R%) of the sensor was determined using Eq. (1)^43^:

1 $${\text{ R\% }} = { }\left| {\frac{{{\text{R}}_{{{\text{CO}}2}} - {\text{ R}}_{{{\text{air}}}} }}{{{\text{ R}}_{{{\text{air}}}} }}} \right|{ } \times { }100$$

Here, R_CO2_ is the sensor response after a predetermined exposure time to CO_2_, and R_air_ is the sensor response in normal air under the same pressure and temperature conditions^24^. As the ZnO thin film was progressively doped, its sensitivity and gas response increased, as evident in Fig. 8, with the rising gas flow rate. In particular, when measuring the response of pure ZnO in Fig. 8a, the values remained low, increasing from 0.6% to 1.6% as the flow rate was raised from 50 to 200 SCCM. However, the response values of ZnO: 2.0 at% La and ZnO: 4.0 at% La exhibited significant enhancement, increasing from 35.99% to 58.36% and from 37.22% to 114.22%, respectively. This improvement can be attributed to the increase in surface roughness and sensitivity^25^. Figure 8 demonstrates that the ZnO: 4.0 at% La sensor displayed the highest sensitivity and response to CO_2_ gas, owing to its pronounced surface roughness, along with the quickest response and recovery time. The response time is the duration required for the relative resistance change to reach 90% of the value at steady state after CO_2_ exposure^44^. From the linear fitted sensor response vs. CO_2_ flow rate in Fig. 8b, the slope (flow rate sensitivity) was calculated to be 0.54 SCCM^−1^. This linear relationship is beneficial for practical gas-sensing applications^45^. Figure 8c depicts the relationship between the estimated response time of the ZnO sensors and the gas flow rate. On the other hand, the recovery time, which is the duration needed to recover a resistance that is 10% higher than the initial value, is illustrated in Fig. 8d. For a flow rate of 200 SCCM, the response time of pure ZnO was 450 s, while ZnO: 2.0 at% La demonstrated a response time of 84 s, and ZnO: 4.0 at% La exhibited the lowest response time of 24.4 s. Similarly, at the same flow rate, the recovery time of pure ZnO was 321 s; ZnO: 2.0 at% La showed a recovery time of 150.44 s, and ZnO: 4.0 at% La had the lowest recovery time of 44 s. These results indicate that increasing the doping percentage led to a reduction in the response and recovery time, with the optimal doping concentration being 4%. This enhancement can be attributed to the film's increased surface roughness, which positively influences its responsiveness and recovery to CO_2_ gas^24^.

#### Long term stability, detection limit, the limit of quantification, and signal to noise ratio

**Figure 9:**
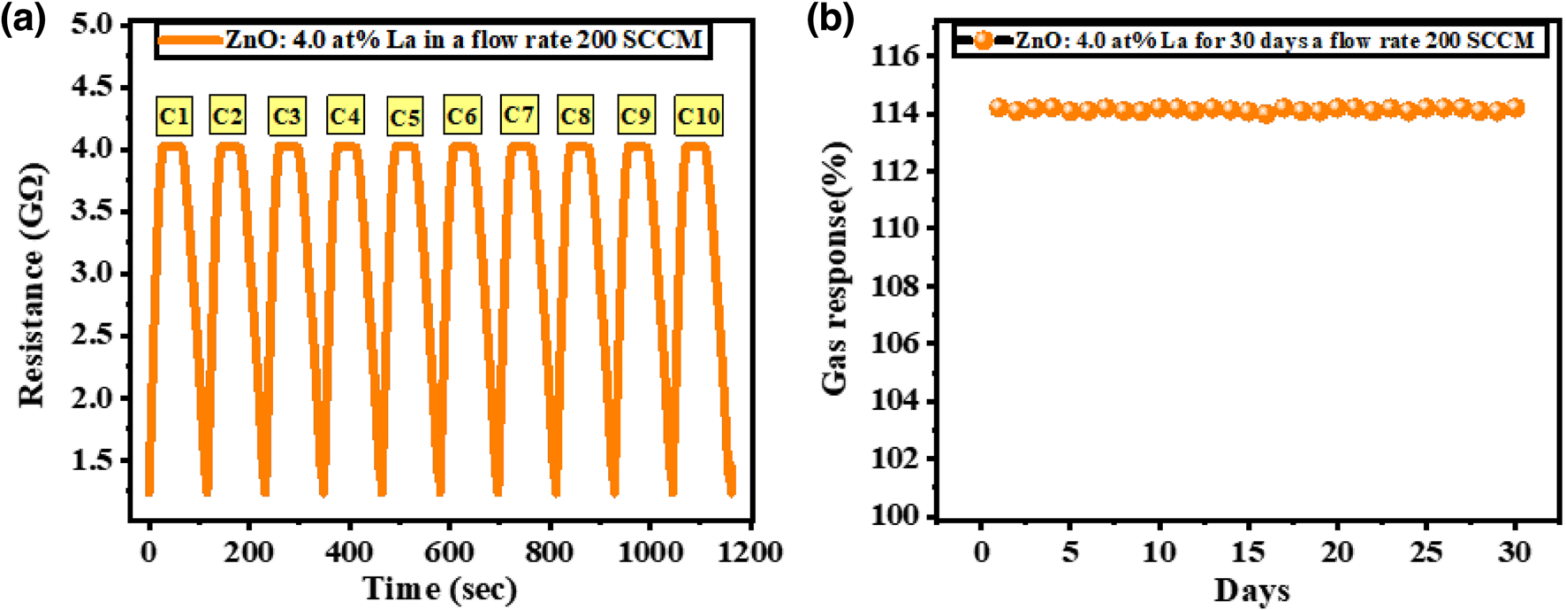
(**a**) Repeatability, and (**b**) Reusability of the ZnO: 4.0 at% La thin film at RT and 30% RH.

A gas sensor's ability to retain its sensitivity over time is crucial to its usefulness in the field. As shown in Fig. 9a, the response of the ZnO: 4.0 at% La sensor was tested after it was exposed to 200 SCCM CO_2_ with syntic air for 10 on/off cycles at RT and 30% RH. As was demonstrated, virtually no variation occurs, indicating high repeatability. Figure 9b reveals that over the course of a month, the ZnO: 4.0 at% La sensor was exposed to 200 SCCM CO_2_ with syntic air at a rate of once per day at RT. The sensor's initial sensitivity was virtually unaffected by the thirty days of continuous testing, as evidenced by the data in this figure. As a result, it was established that the sensor's construction material was of excellent quality and suitable for continuous use. The sensor's sensitivity value stayed close to 114% during the whole experiment. This proved consistent repeatability since no drift in the baseline was detected. And the results clearly demonstrate that the constructed sensor exhibits excellent reversibility and repeatability when exposed to carbon dioxide gas.

Using the sensor's standard deviation (SD) in sensor response and the slope of the straight section when exposed to low concentrations, we were able to determine the sensor's detection limit (DL) using the following Eq. (2)^46^.

2 $${\text{DL}} = \left( {{3} \times {\text{SD}}} \right)/{\text{Slope}}$$

The DL for pure ZnO was 201 SCCM as the slope was 0.0123 GΩ/sec and the SD was 0.828, but for ZnO: 2.0 at% La DL was determined to be 16.2 SCCM, while the slope was 0.05 GΩ/sec and the SD was 0.27. On the other hand, with great performance, the detection limit for ZnO: 4.0 at% La was determined to be 0.526 SCCM, while the slope was 1.17 GΩ/sec and the standard deviation was 0.07. The DL results indicate that the ZnO: 4.0 at% La film is the most effective one since it has the lowest detection limit^41^.

The sensor limit of quantification (QL), representing the minimum number of CO_2_ molecules detectable by the sensor, was successfully determined. The QL is defined as follows in equation (3)^47^:

3 $${\text{QL}} = \left( {{1}0 \times {\text{SD}}} \right)/{\text{Slope}}$$

It has been determined that the QL for pure ZnO is 673, and for ZnO: 2.0 at% La is 54, while for ZnO: 4.0 at% La is 0.59. According to the values of QL, the ZnO: 4.0 at% La sensor is the most effective one because it has the lowest value of QL.

The signal-to-noise ratio (S/N) was determined using the following Eq. (4) from the height of the peak(H) at low concentration and the FWHM(h)^43^, which 6.05, 5.55, and 1.546 for pure ZnO, ZnO: 2.0 at% La, and ZnO: 4.0 at% La, respectively.

4 $$\frac{{\text{S}}}{{\text{N}}} = \frac{{2{\text{H}}}}{{\text{h}}}$$

#### Effect of humidity and sensor selectivity

**Figure 10:**
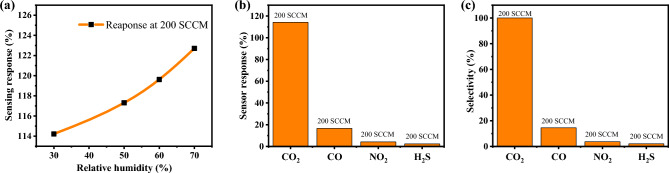
(**a**) Selectivity of the ZnO: 4.0 at% La at RT and 30% RH, and (**b**) Effect of humidity on sensing response at RT.

The impact of humidity on the sensing response was investigated by subjecting the RH to an increase of 30–70% in a humidity-controlled chamber while introducing 200 SCCM of CO_2_ gas at RT, as depicted in Fig. 10a. When subjected to a CO_2_ flow rate of 200 SCCM, the constructed sensor exhibited a modest increase in dynamic sensing response as the RH was raised by 30%, 50%, 60%, and 70%, resulting in values of 114.22%, 117.31%, 119.62%, and 122.71%, respectively. The presence of humid air leads to the formation of OH^−^ ions on the surface of the sensing material, which serve as catalysts for the reaction of CO_2_ adsorption. The obtained outcome suggests that humidity significantly influences the adsorption of CO_2_ by sensing materials. When the humidity level exceeds 75%, the sensing response diminishes as a result of the sensing material being fully covered by water molecules, which reduces the available reaction sites for CO_2_ interaction^48^. Figure 10b depicts the selectivity investigation performed on the ZnO: 4.0 at% La. The results indicate that the sensor exhibits a relatively low response of 16.6%, 4.2%, and 2.3% to the 200 SCCM flow rate of interference gases such as carbon monoxide, nitrogen dioxide, and hydrogen sulfide, respectively. In comparison, the sensor demonstrates a much higher response of 114.22% to the 200 SCCM CO_2_ gas. The result shows that the ZnO: 4.0 at% La sensor made for CO_2_ gas is much more sensitive to CO_2_ than to other gases that could interfere, all at RT.

The most common method for testing sensor selectivity is to expose the device under test to multiple gases and quantify the sensor response ratio for each gas, as shown in Eq. (5):

5 $${\text{Selectivity }}\;\left( {\text{\% }} \right) = \frac{{{\text{R}}_{{{\text{other}}\;{\text{ gas}}}} }}{{{\text{ R}}_{{{\text{target}}\;{\text{ gas}}}} }}{ } \times 100$$

Figure 10b demonstrates that the sensor responded more strongly to CO_2_ than to the other gases tested (R_CO2_ > R_CO_ > R_N2_ > R_H2S_), and Fig. 10c illustrates the selectivity for various gases based on CO_2_; the percentages for CO, NO_2_ and H_2_S are 14.53, 3.67711, and 2.01, respectively.

#### Gas sensing mechanism

**Figure 11:**
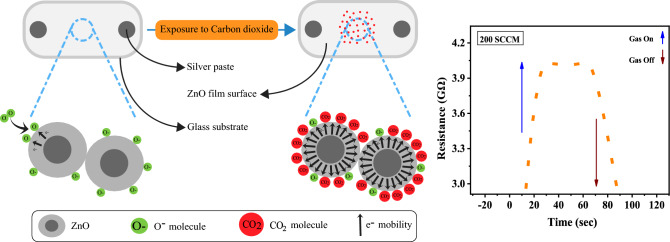
Gas sensing mechanism of the ZnO thin films.

The principal gas sensing mechanism of ZnO is based on the adsorption of CO_2_ molecules on its surface, resulting in a change in the surface electrical resistance^49^. The gas sensing mechanism of carbon dioxide (CO_2_) with ZnO thin films doped with lanthanum (La) involves a series of interconnected steps that enable the detection of CO_2_ concentrations. When ZnO film is exposed to oxygen (O_2_) in the air, oxygen molecules are adsorbed onto the surface of the doped ZnO thin film, leading to the transfer of electrons from the conduction band (CB) and the introduction of reactive oxygen species (O^−^, O^2−^) and the creation of oxygen vacancies within the ZnO lattice. These vacancies act as electron donors, providing excess electrons to the doped ZnO thin film and enhancing the mobility of charge carriers (electrons), resulting in increased electrical conductivity or reduced resistance of the material. As CO_2_ gas is introduced into the environment, it chemisorbs onto the surface of the doped ZnO thin film, capturing some of the extra electrons from the ZnO lattice and forming carbonate compounds like (CO_3_)^2−^. This interaction reduces the number of available charge carriers, leading to a decrease in electrical conductivity or an increase in resistance, as ZnO is an n-type semiconductor. The change in electrical resistance serves as a gas-sensing signal, indicating the presence and concentration of CO_2_ in the surrounding environment. Moreover, the incorporation of lanthanum in the ZnO thin film enhances its gas-sensing performance due to its higher surface area, which provides more pathways for CO_2_ diffusion and offers abundant active sites for increased gas absorption. This property makes La-doped ZnO a promising material for highly sensitive and selective CO_2_ gas sensors^48,49^. Figure 11 shows the gas sensing mechanism of the ZnO thin films.Table 2 shows detailed results that compare the ability of ZnO sensors to detect CO_2_ gas as reported in previous research with the results of this study in this field.

**Table 2:** A comprehensive comparison of the CO_2_ gas detecting characteristics obtained from prior studies on CO_2_ sensors using ZnO with the current work.

Nanostructured	Temperature (°C)	R (%)	Concentration or flow rate	t_response_	t_recovery_	Ref
CuO/ZnO (C/Z)	375 °C	47%	2500 ppm	–	–	^13^
12h—ZnO	RT	28.7%	180 SCCM	11.4 min	3.8 min	^43^
2 mol % W-doped ZnO	450 °C	98%	1000 ppm	–	–	^50^
ZnO/CNTs	RT	22.4%	150 SCCM	82.5 s	23 s	^16^
ZnO nanomaterial	350 °C	–	100 ppm	4 min	5 min	^51^
ZnO: 4.0 at% La	RT (30 °C)	114.22%	200 SCCM	24.4 s	44 s	This work

## Conclusion

In this study, we focused on making and studying ZnO films doped with La for gas sensing at RT. La-doping affected the film's structure, optics, and gas sensing properties. The films had a hexagonal shape and the best growth in the (002) direction. With increasing La content, the crystallite size decreased, confirming successful La incorporation. The La-doped ZnO films maintained high transparency (> 80%) and had modified optical properties, blocking more UV and narrowing the band gap (Eg) from 3.275 to 3.125 eV. Regarding gas sensing, we tested the sensors at RT (30 °C) and 30% RH using various CO_2_ gas flow rates mixed with air. The ZnO: 4.0 at% La sensor exhibited the highest gas response of 114.22% at a flow rate of 200 SCCM of CO_2_ gas. It also showed fast response and recovery times of 24.4 seconds and 44 seconds, respectively. These impressive results indicate its potential for detecting CO_2_ gas in industrial applications. Additionally, we investigated other factors like humidity, selectivity, reusability, repeatability, detection limit, and limit of quantification. Overall, this research highlights the promise of La-doped ZnO films as effective gas sensors at RT, contributing to enhanced environmental and health safety measures in various industries. Further research can optimize doping concentrations and fabrication techniques to further enhance gas sensing performance.

### Supplementary Information

Supplementary Figures.

## Data Availability

The data presented in this study are available on request from the corresponding author.
