# Supplementary material for: Design of high-sensitivity La-doped ZnO sensors for CO2 gas detection at room temperature
Source: Sci Rep. 2023 Oct 26;13:18398. doi: 10.1038/s41598-023-45196-y (PMC10603051; doi:10.1038/s41598-023-45196-y)
Supplement: Supplementary file 1 — Supplementary Figures. [file 41598_2023_45196_MOESM1_ESM.docx]

Electronic Supplementary Material

**Design of High-Sensitivity La-Doped ZnO Sensors for CO_2_ Gas Detection at Room Temperature**

Khaled Abdelkarem^1*^(K.A.), Rana Saad^1^(R.S.), Adel M. El Sayed^2^(A.M.S.), M. I. Fathy^1^(M.I.), Mohamed Shaban^1,3**^(M.S.), and Hany Hamdy^1^(H.H.)

^1^Nanophotonics and Applications (NPA) Lab, Department of Physics, Faculty of Science, Beni-Suef University, Beni-Suef 62514, Egypt; oldfighter.khaled123@gmail.com (K.A.); ranasaad811@gmail.com (R.S.); ismail.moataz@yahoo.com (M.I); mssfadel@aucegypt.edu (M.S.); hshamdy@hotmail.com (H.H.)

^2^ Physics Department, Faculty of Science, Fayoum University, El Fayoum 63514, Egypt; ams06@fayoum.edu.eg (A.M.E)

^3^Department of Physics, Faculty of Science, Islamic University in Almadinah Almonawara, Almadinah Almonawara, 42351, Saudi Arabia; mssfadel@aucegypt.edu (M.S.)

^*^ email: oldfighter.khaled123@gmail.com

^**^ email: mssfadel@aucegypt.edu


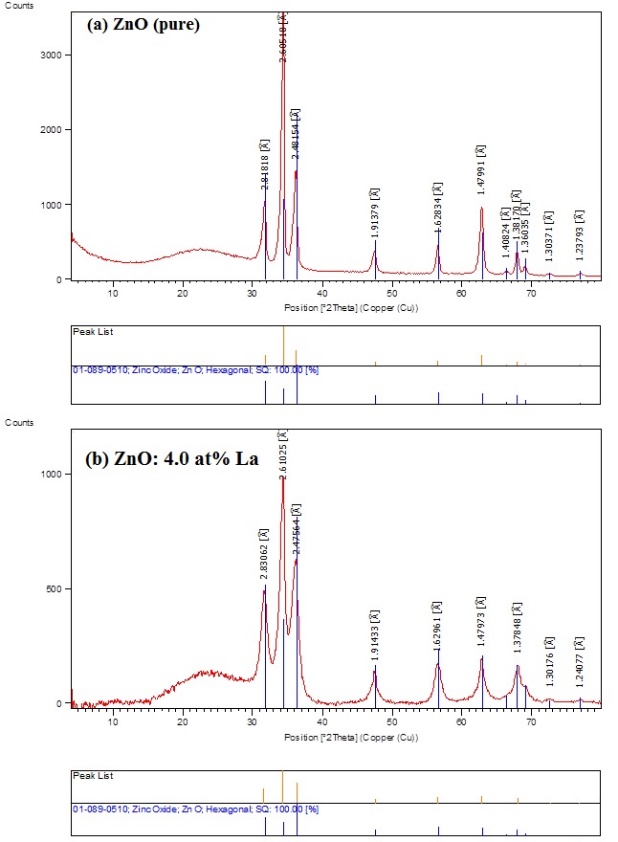


S1: The XRD data of pure and ZnO: 4.0 at% La according to JCPDS 01-089-0510


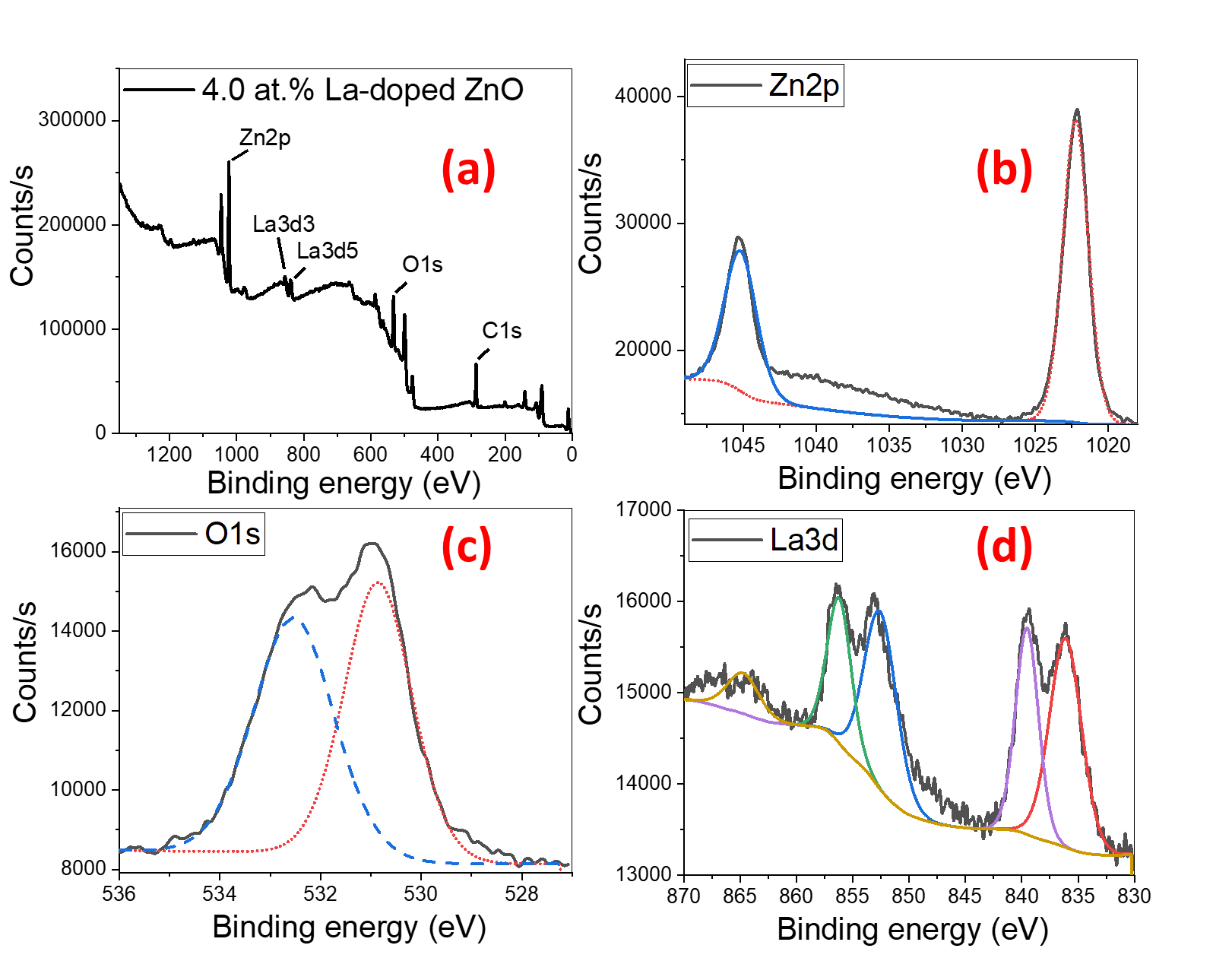


Figure S2: Thickness measurement for (a) Pure and (b) 2% La-doped ZnO from the SEM analysis.


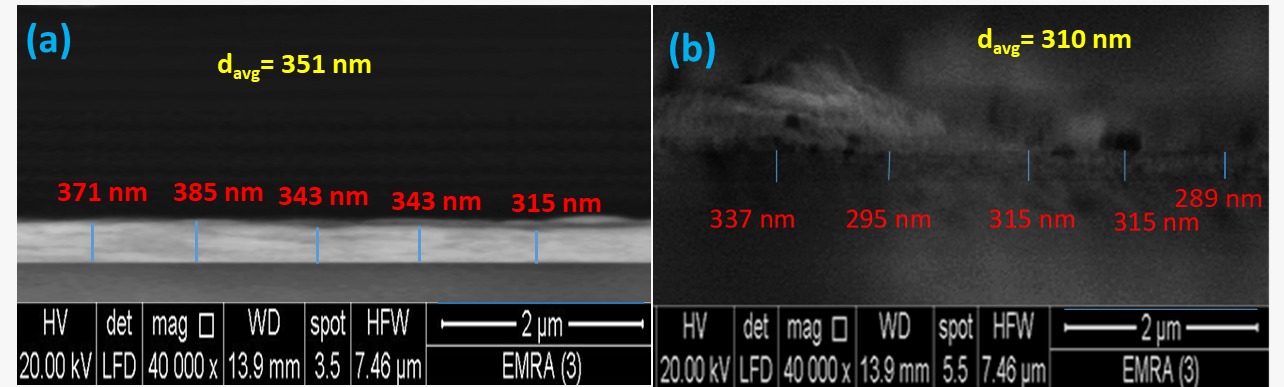


Figure S3: Thickness measurement for (a) Pure and (b) 2% La-doped ZnO from the SEM analysis.
